# Cooperative membrane association as a mechanistic origin of synergistic antimicrobial peptide activity

**DOI:** 10.1039/d6cb00084c

**Published:** 2026-05-20

**Authors:** Angela Medvedeva, Anatoly B. Kolomeisky

**Affiliations:** a Department of Chemistry, Rice University Houston Texas 77005 USA tolya@rice.edu; b Center for Theoretical Biological Physics, Rice University Houston Texas 77005 USA; c Department of Chemical and Biomolecular Engineering, Rice University Houston Texas 77005 USA; d Department of Physics and Astronomy, Rice University Houston Texas 77005 USA

## Abstract

Antimicrobial peptides (AMPs) are central components of the innate immunity system that can be found in almost all living organisms. They are promising alternatives to conventional antibiotics due to their broad-spectrum activity and reduced susceptibility to resistance. Experimental studies have shown that combinations of AMPs can act synergistically, achieving enhanced antibacterial efficacy at lower total concentrations in combination than individually. However, despite its prevalence, AMP synergy has until recently been lacking a unifying mechanistic and predictive framework. In this review, we combine the latest theoretical, computational, and experimental advances to present a novel quantitative framework that views AMP synergy as a consequence of cooperative membrane association. Chemical-kinetic models of AMPs association to bacterial membranes show that favorable intermolecular interactions between different AMP species accelerate their binding, enhancing antibacterial activity. Within this framework, an effective interaction parameter, Δ*E*, emerges as a quantitative descriptor of cooperativity linking microscopic interactions to macroscopic synergy metrics such as minimal inhibitory concentrations (MIC). Extensions of this approach rationalize the enhanced efficacy of heterogeneous multi-AMP mixtures and clarify the specific case of AMPs associating to each other as hetero-oligomers before binding to the bacterial membranes. Complementary statistical and machine-learning analyses further demonstrate that synergistic AMP combinations are characterized by physicochemical complementarity rather than similarity, enabling prediction of synergy from sequence-derived features. The review demonstrates that AMP synergy can be quantitatively described and potentially rationally designed using a combined chemical-kinetic and statistical machine-learning approach, providing a foundation for systematic development of effective and resistance-resilient multi-AMP therapeutics.

## Abstract 2

AMP–AMP combinations are tested through checkerboard assays, and their antibacterial properties are measured. Synergy occurs at AMP concentrations that are shown by striped green circles.

## Graphical abstract

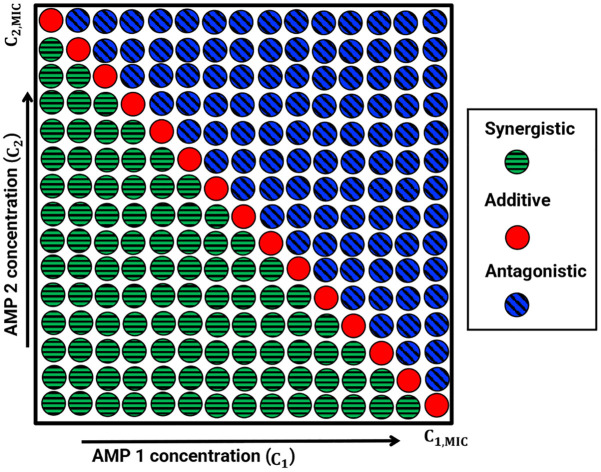

## 1. Introduction

The rapid emergence and global spread of antimicrobial resistance represents one of the most serious threats to modern medicine.^1–5^ Classical antibiotics, which are typically small organic molecules that target specific cellular biochemical pathways, have been successful in eliminating bacteria for decades.^6^ However, their widespread and prolonged use has also led to the rapid evolution of resistant bacterial strains through spontaneous mutations and horizontal gene transfer.^7–11^ As a result, the efficacy of existing antibiotics continues to decline, while the pipeline of new antimicrobial agents remains very limited.^12–14^ These alarming trends have stimulated an intensive search for alternative antibacterial strategies.^15,16^

Antimicrobial peptides (AMPs), also known as host defense peptides, have emerged as promising candidates to complement or replace conventional antibiotics.^17–21^ AMPs are peptides produced by multicellular organisms as part of their innate immunity defense against external infections.^22–25^ They are characterized by a high density of positively charged cationic residues and a substantial fraction of hydrophobic amino acids, typically clustered together, allowing them to act against a broad spectrum of pathogens, including bacteria, fungi, viruses, parasites, and cancer cells.^19,21,26–28^ Importantly, AMPs exhibit selective toxicity: they preferentially target bacterial cells while remaining relatively safe to host tissues.^29–31^ In contrast to antibiotics, bacterial resistance to AMPs is generally less likely, making them attractive candidates for long-term antimicrobial therapies.^32–36^

Despite these advantages, the clinical translation of AMPs faces several challenges. Although resistance to individual AMPs is rare, it is still possible,^37–41^ and long-term AMP exposure can induce toxic side effects, including hemolytic activity and inflammation.^18,33^ Consequently, strategies that minimize dosage while preserving antibacterial efficacy are crucial for development of novel medical antibacterial therapies. One such strategy is the use of combinations of AMPs rather than single peptide agents.

A growing body of experimental evidences demonstrates that combinations of two or more AMPs frequently exhibit synergistic (positive cooperativity) antimicrobial activity, meaning that the total peptide concentration required to inhibit bacterial growth is substantially lower than that needed when each peptide is applied individually.^35,42–45^ Well-established examples include combinations of temporin A and temporin B peptides, which individually display limited antimicrobial activity but when combined with temporin L peptides exhibit strong synergy against Gram-negative bacteria.^46^ Synergistic effects have also been observed in various AMP mixtures targeting planktonic bacteria, biofilms, and antibiotic-resistant strains, as well as in many others multi-peptide cases.^44,47^ However, combinations of AMPs can also be antagonistic (negative cooperativity), with higher concentrations of each AMP type required to eliminate bacteria in combination compared to individually.^48^ There are also additive (neutral cooperativity) cases when bactericidal concentrations are identical for each type of AMP regardless of whether it is alone or in a mixture.^49^

Although AMP cooperativity is now widely recognized as a robust and reproducible phenomenon, with the synergy being quantified empirically, the underlying mechanisms are less clear.^44,45^ In particular, there is no broadly accepted picture that explains why certain AMP combinations are synergistic while others are not, how synergy scales with the number and type of peptides involved, or which molecular parameters control the transition between synergy, additivity, and antagonism. Moreover, predictive approaches capable of identifying specific synergistic AMP combinations against specific pathogens remain quite limited.

We discuss here a novel theoretical framework that shows how synergistic antimicrobial activity in AMP combinations might emerge from cooperative membrane association. It is based on a hypothesis that effective intermolecular interactions between different AMP species can accelerate their collective association with bacterial membranes, leading to faster bacterial killing. This cooperativity is quantified using effective chemical-kinetic models that could be applied to experimentally measurable quantities, such as bactericidal concentrations of each AMP type alone *versus* in combination. Furthermore, the same mechanistic framework provides a foundation for predicting specific synergistic AMP combinations based on physicochemical complementarity using statistical and machine-learning methods.

## 2. Antimicrobial peptides

### 2.1. Structural and physicochemical features of AMPs

Antimicrobial peptides are relatively short biopolymeric molecules, typically consisting of approximately 10–50 amino acids.^26,50,51^ A defining feature of most AMPs is their predominantly cationic nature, with net charges commonly ranging from +2 to +11,^52–54^ arising from a high abundance of positively charged lysine and arginine residues.^55–57^ It is important to note that not all AMPs are cationic. Certain AMPs are anionic and contain predominantly negatively charged residues.^58,59^ Furthermore, AMPs usually contain a substantial number of hydrophobic amino acids, approximately 50% of the sequence composition, and hydrophobic residues are tend to cluster together.^60^ These physicochemical characteristics give rise to amphipathic architectures, in which cationic and hydrophobic residues are spatially segregated either along the primary sequence or upon secondary structure formation.^61^ Amphipathicity also plays an important role in AMPs functioning as antibacterial agents.

AMPs exhibit significant diversity in sequence, length, secondary structure (α-helical, β-sheet, mixed, or disordered), and physicochemical profiles.^62^ Importantly, numerous studies have shown that antimicrobial activity correlates more strongly with global physicochemical properties, such as charge density, hydrophobicity, amphipathicity, and flexibility, rather than with precise amino acid sequence or structural motifs.^63–65^ These observations suggest that AMP functions may be governed by universal collective physicochemical properties, which could partially explain the high variability in AMP types and structures.

### 2.2. Membrane selectivity

Many AMPs demonstrate the ability to selectively target bacterial membranes while sparing the membranes of host cells. This selectivity arises primarily from fundamental differences in membrane composition between prokaryotic and eukaryotic organisms.^29,66^ Bacterial membranes are enriched in negatively charged phospholipids, creating a strong electrostatic attraction for cationic peptides. In contrast, eukaryotic cell membranes are largely zwitterionic and contain high levels of cholesterol, which significantly reduces peptide binding and membrane disruption. In addition, electrostatic interactions may drive the initial association of AMPs with bacterial membranes, while hydrophobic interactions facilitate further peptide insertion and membrane perturbation.^29^ These physicochemical principles underlie the broad-spectrum activity and selectivity of AMPs and form the basis for most mechanistic models of AMPs functioning.^17–21^

### 2.3. Mechanisms of bacterial killing

It is known that AMPs exhibit a variety of antibacterial mechanisms. However, the membrane-associated processes typically dominate.^27,30,67,68^ After binding to the bacterial membrane, AMPs can accumulate on the surface, possibly aggregate and induce membrane permeabilization through pore formation. This leads to leakage of cellular contents, loss of membrane integrity, and ultimately bacterial cell death.

Although some AMPs can also act on intracellular targets, such as nucleic acids or metabolic enzymes, these pathways are relatively rare and still require prior membrane association.^17,24^ Consequently, regardless of the downstream killing mechanism, membrane binding can be considered as a first crucial step in AMP antimicrobial activity for all different scenarios of bacterial elimination. This observation motivates a theoretical proposal that the efficiency of bacterial killing might be primarily controlled by the dynamics of AMP association with the membrane.^69^

## 3. Experimental observations of synergy for AMP combinations

### 3.1. Definitions and quantitative parameters of AMPs functioning

The antibacterial efficacy of AMPs is commonly quantified using a concept of minimal inhibitory concentration (MIC), defined as the lowest concentration of peptide required to stop bacterial growth.^70^ This is a property that describes the action of a single individual AMP. For AMP combinations, synergy (cooperativity) is typically assessed using a so-called fractional inhibitory concentration (FIC) parameter, which measures how much the MIC of each component is reduced when peptides are applied together relative to their individual MIC values:^71^1
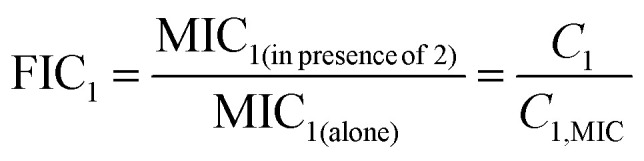
2
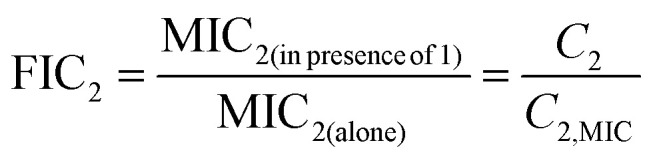

3 FIC = FIC_1_ + FIC_2_

**Fig. 1:**
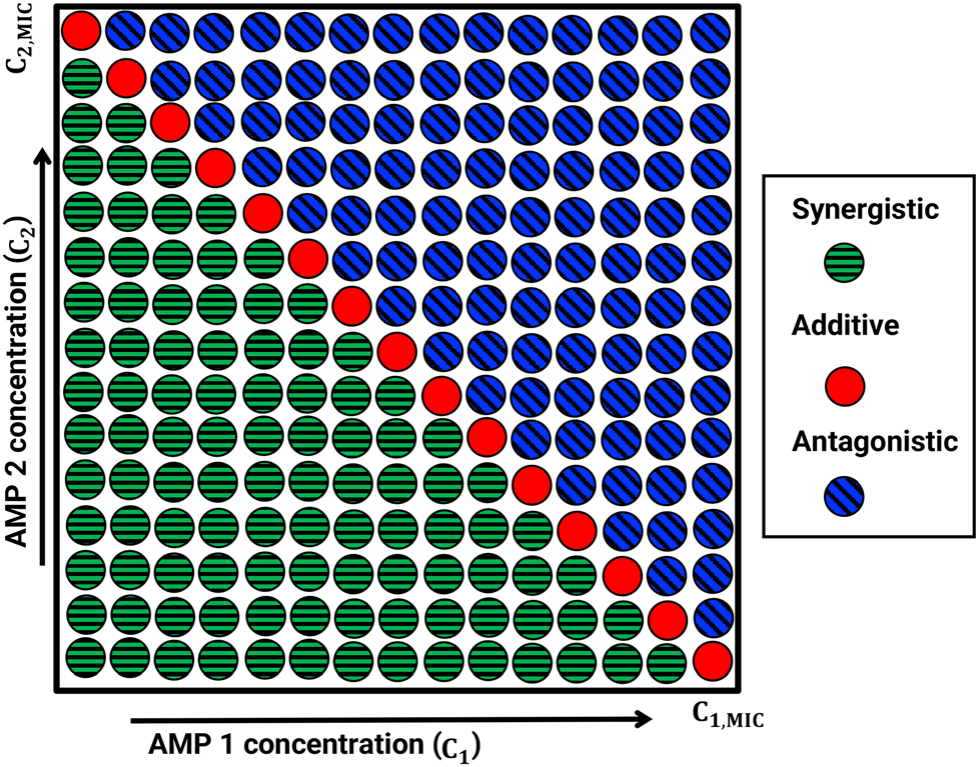
Illustration of a checkerboard assay through which AMP–AMP combinations are tested and their antibacterial properties are measured. Synergy occurs at AMP concentrations that are shown by striped green circles. Additivity corresponds to no cooperativity and it is shown by solid red circles. Antagonism with negative cooperativity is shown by hatched blue circles.

Using FIC parameters it is convenient to classify combination effects. AMP combinations with FIC values below 1 can be considered as synergistic (positive cooperativity), values near 1 indicate additive effects (no cooperativity), and values greater than 1 correspond to antagonism (negative cooperativity).^69,72^ These metrics provide a convenient standardized experimental approach for comparing antimicrobial activities across different peptides, combinations, and bacterial species. Cooperativity is commonly probed in experiments by using a checkerboard assay in which the concentration of one AMP is kept constant while the other is increased in small fractions, until the combination is effective in inhibiting bacteria growth. The schematic view of such experimental measurements is presented in Fig. 1. This is a standard way of testing synergy among any sets of antimicrobials.^73^

### 3.2. Prevalence of synergistic effects

Synergistic interactions between AMPs have been observed across a wide range of systems.^45^ Numerous studies reported strong synergy in two-peptide combinations, often involving peptides with distinct physicochemical properties or biological origins.^74–77^ Beyond combinations of two different AMP species, experiments also demonstrated that multi-peptide mixtures frequently exhibit even stronger synergy than two-component systems, despite having similar total peptide concentrations.^44^ These effects have been observed in different bacterial species, including Gram-positive and Gram-negative strains^78^ as well as in biofilm-forming and antibiotic-resistant populations.^79^ The prevalence of synergistic effects across different contexts suggests that AMP synergy is not an isolated phenomenon but a common and biologically relevant feature of antimicrobial defense for all organisms. These observations also indicate that there are possibly universal mechanisms of antibacterial action for AMP combinations.

Despite extensive experimental evidence, existing mechanistic interpretations of AMP synergy remain quite limited.^80,81^ Additivity can be assumed as a baseline and deviations from additivity have been attributed to unspecified or system-specific effects. Synergy have been quantified but not mechanistically explained, and key parameters controlling cooperative behavior also have not been identified. Furthermore, available datasets are sparse, heterogeneous, and often organism-specific, making it difficult to generalize empirical observations or develop predictive principles. As a result, there is a clear need for mechanistic quantitative frameworks that connect measurable physicochemical and kinetic parameters to observed cooperativity, and that can also rationalize why certain combinations are synergistic while others are not.

## 4. Cooperative membrane association: a chemical-kinetic framework

### 4.1. Membrane adsorption as a chemical-kinetic process

A central idea underlying the chemical-kinetic approach is that membrane-active AMPs can eliminate bacteria only after associating with the bacterial membrane, and therefore the overall antibacterial efficacy can be controlled by the dynamics of membrane association.^69,82^ The presence of different types of AMPs accelerate the overall association to cellular membranes, which leads to faster bacterial killing. The processes of bacterial growth, AMP association and bacterial killing are viewed as as set of chemical reactions.^69,82^ In this description, the bacterial population is time-dependent. In the absence of AMPs, bacteria grow exponentially through cell divisions, while in the presence of AMPs their growth starts to slow down and eventually bacterial concentration decreases due to AMP-mediated killing. The minimal inhibitory concentration (MIC) corresponds to the condition in which the number of bacterial cells stops increasing when growth and killing balance each other.

To be more specific, consider the action of AMP molecules of one type with concentration *C*(*t*) on bacterial species with concentration *B*(*t*) at time *t*. Then the processes in the system can be described as two “chemical” reactions,4
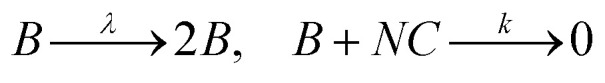
where *N* is the number of AMP molecules needed to eliminate a single bacterial cell, *λ* is the rate constant for bacterial growth and *k* is the rate constant of bacterial killing by AMPs. In experimentally relevant conditions, *N* ≫ 1, *N* ∼ 10^4^–10^8^ peptides are required to kill bacteria.^83^ The corresponding chemical-kinetic equation describes the temporal evolution in the system as5
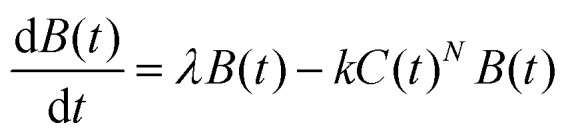


In this equation, the first term is for bacterial growth, while the second term is responsible for bacterial elimination. This picture allows to estimate the conditions at which the bacterial growth stops 
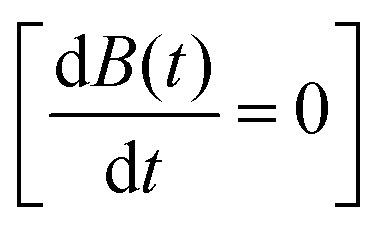
, which corresponds to MIC,6
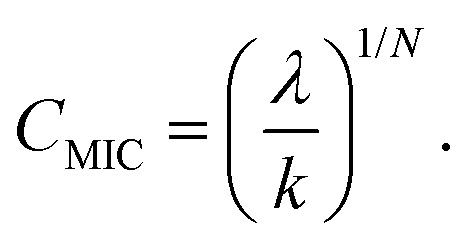


The theoretical approach can be easily generalized for combinations of two types of AMP molecules with concentrations *C*_1_(*t*) and *C*_2_(*t*).^69,82^ In this system, the chemical equations can be written as7
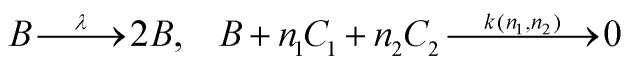
where *k*(*n*_1_,*n*_2_) is an effective rate constant for association, and *n*_1_ and *n*_2_ are numbers of AMPs of type 1 and 2, respectively, with *n*_1_ + *n*_2_ = *N*. The corresponding chemical-kinetic equation is8
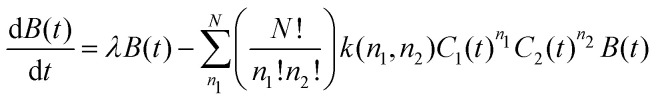


In this expression, 
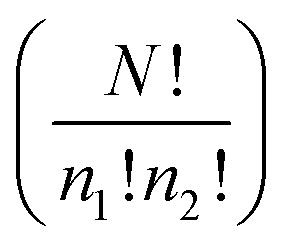
 is the number of of possible ways *n*_1_ AMP molecules of type 1 and *n*_2_ molecules of type 2 can associate to bacterial membrane. The conditions for which bacterial growth stops are again evaluated by taking 
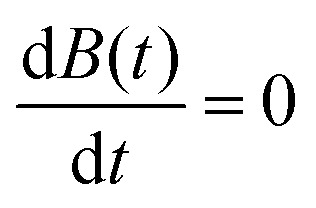
.^69^ This yields the following relation,9
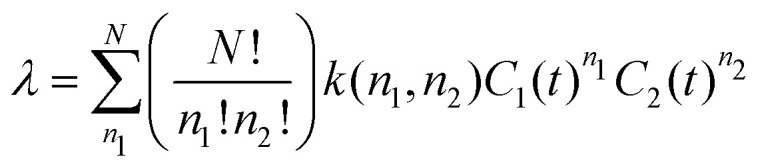


The analysis further simplifies in the realistic limit of *N* ≫ 1 when it can be argued that dominating associations occur for *n*_1_∼ *n*_2_∼ *N*/2 when the number of different AMPs is comparable to each other.^69^

For understanding the microscopic origin of cooperativity for enhanced bacterial killing by a mixture of different types of AMPs, a central role in theoretical analysis is played by a parameter *k*(*n*_1_,*n*_2_) [see eqn (8)], which is defined as an effective rate constant for the process of AMPs association to bacterial membrane. It can be presented as10
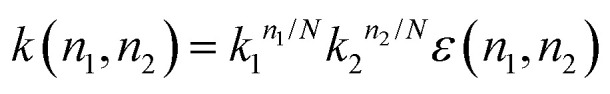
where *k*_1_ and *k*_2_ are the rate constants of binding of only AMPs of type 1 or 2 respectively, and *ε*(*n*_1_,*n*_2_) is a phenomenological parameter that reflects the degree of cooperativity in the system. For *ε*(*n*_1_,*n*_2_) > 1 a synergy in AMPs actions against bacteria is expected, *ε*(*n*_1_,*n*_2_) = 1 corresponds to the additive case without cooperativity, and *ε*(*n*_1_,*n*_2_) < 1 describes the antagonistic situations.

Within this framework, cooperativity is quantified through an effective interaction energy parameter, Δ*E*,^69,82^ which represents the effective contribution of intermolecular interactions between different AMP species per unit membrane-bound AMP molecule. It is introduced by assuming that the phenomenological parameter *ε*(*n*_1_,*n*_2_) can be written as11
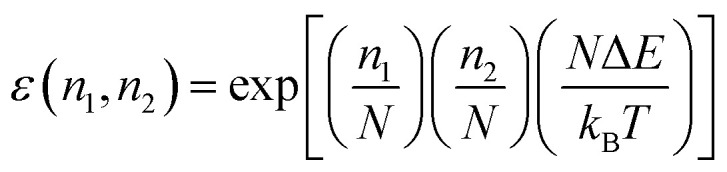


The physical meaning of this relation can be explained using the following arguments. The interaction will only occur if two neighboring membrane-bound AMP molecules are of different types, and the product 
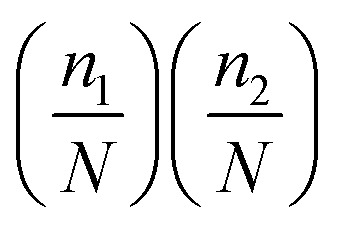
 quantifies the probability of such events. The coefficient *N* reflects the fact that there are *N* AMP molecules of any type are needed to kill a single bacterial cell. This picture additionally assumes that bound AMPs of both types are uniformly distributed along the membrane.

**Fig. 2:**
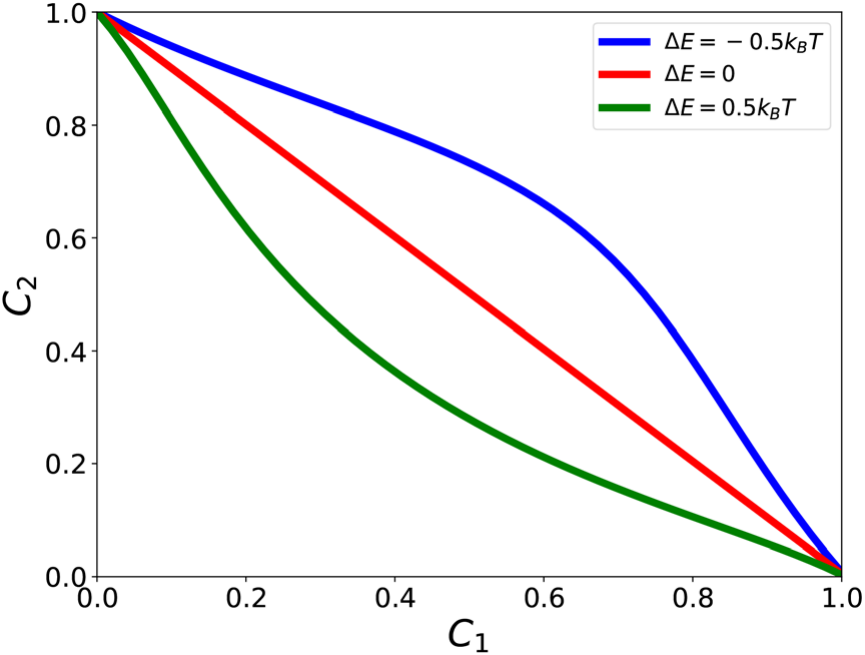
Evaluating the concentrations of AMPs of type 1 and 2 that stop bacterial growth for different interaction energies Δ*E*. The concentrations *C*_1_ and *C*_2_ are normalized with respect to the corresponding MIC of single components. For calculations, the following parameters have been used: *N* = 10, *λ* = 1/20 min^−1^, *k*_1_ = 0.75*λ* and *k*_2_ = 0.5*λ*. The figure is adopted with permission from ref. 69.

**Fig. 3:**
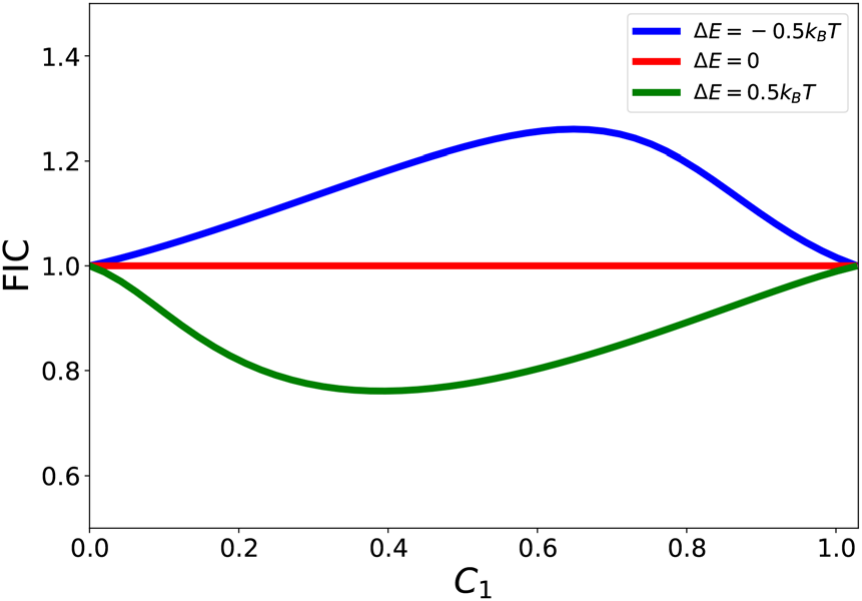
Evaluating FIC parameters for the system with AMPs of type 1 and 2 that stop bacterial growth for different interactions energies Δ*E*. For calculations, the following parameters have been used: *N* = 10, *λ* = 1/20 min^−1^, *k*_1_ = 0.75*λ* and *k*_2_ = 0.5*λ*. The figure is adopted with permission from ref. 69.

The parameter Δ*E* explicitly evaluates the degree of cooperativity for a combination of AMPs to eradicate bacterial species.^69,82^ This is because it affects the effective rate constant of association as shown in eqn (10) and (11). In particular, mixtures with Δ*E* > 0 exhibit faster association than single-component peptides, whereas Δ*E* < 0 slows association relative to the single-component reference. Δ*E* = 0 reflects no influence of cooperativity on speed of membrane association. Fig. 2 shows the results of theoretical calculations for the system of two different AMPs eradicating bacterial cells. The antibacterial process can be also described by estimating FIC parameters as illustrated in Fig. 3. One can see that the theoretical framework predicts that even very modest interactions (±0.5*k*_B_*T*) can have significant effect on cooperative dynamics of AMPs in bacterial eradication. These results will be further described in Section 4.3.

### 4.2. Possible origin of cooperativity

A key mechanistic hypothesis of this theoretical method is that cooperativity arises because the presence of one AMP type can stimulate the association of another type with the membrane, leading to faster binding compared to the case where that second AMP acts alone.^84,85^ In this picture, it is important that peptides of only different types of species can interact during the binding process to accelerate the association.

These cross-species interactions may be direct, such as attractive peptide–peptide contacts that stabilize mixed oligomers, or indirect, mediated by changes in the membrane environment induced by the first peptide.^80,86^ For example, the initial binding of one AMP can locally perturb lipid packing, induce membrane thinning or curvature, or modify surface charge distributions, thereby lowering the energetic barrier for insertion or adsorption of other AMPs. In addition, mixed assemblies of different peptides may stabilize pore-like structures or other membrane-disruptive configurations more effectively than single-component systems. As a result, these favorable interactions lower the effective free energy of membrane-bound mixed states and increase the overall association rate, so that the mixture accumulates on the membrane more rapidly than individual components. This accelerated accumulation allows the system to reach the critical threshold number of bound peptides required for bacterial killing more quickly, thereby reducing the minimal inhibitory concentration and producing synergistic behavior.^78,87^

Conversely, the same framework also implies that if interactions between different AMP species are unfavorable, the opposite outcome–antagonism–can arise. In such cases, one peptide may hinder the binding of another through mechanisms such as competition for membrane binding sites, electrostatic screening that reduces membrane affinity, membrane rigidification that increases insertion barriers, structural incompatibility between peptide assemblies, or sequestration into nonproductive aggregates in solution. These effects effectively raise the free energy of mixed membrane-bound configurations and slow down the overall association kinetics. As a consequence, the accumulation of peptides on the membrane is delayed, higher concentrations are required to reach the bactericidal threshold, and the combined activity appears weaker than that of the individual components.

### 4.3. Evaluating synergy from experimental observations

The chemical-kinetic framework can be directly used to evaluate cooperativity from experimental observations. In the absence of intermolecular interactions (Δ*E* = 0), the theory predicts additivity, corresponding to FIC = 1. Introducing positive cooperativity (Δ*E* > 0) corresponds to FIC <1, reflecting synergy, while negative cooperativity (Δ*E* < 0) corresponds to FIC > 1, reflecting antagonism. Thus, the theoretical framework provides a direct mapping from microscopic cooperative interactions to experimentally measurable synergy metrics.

**Fig. 4:**
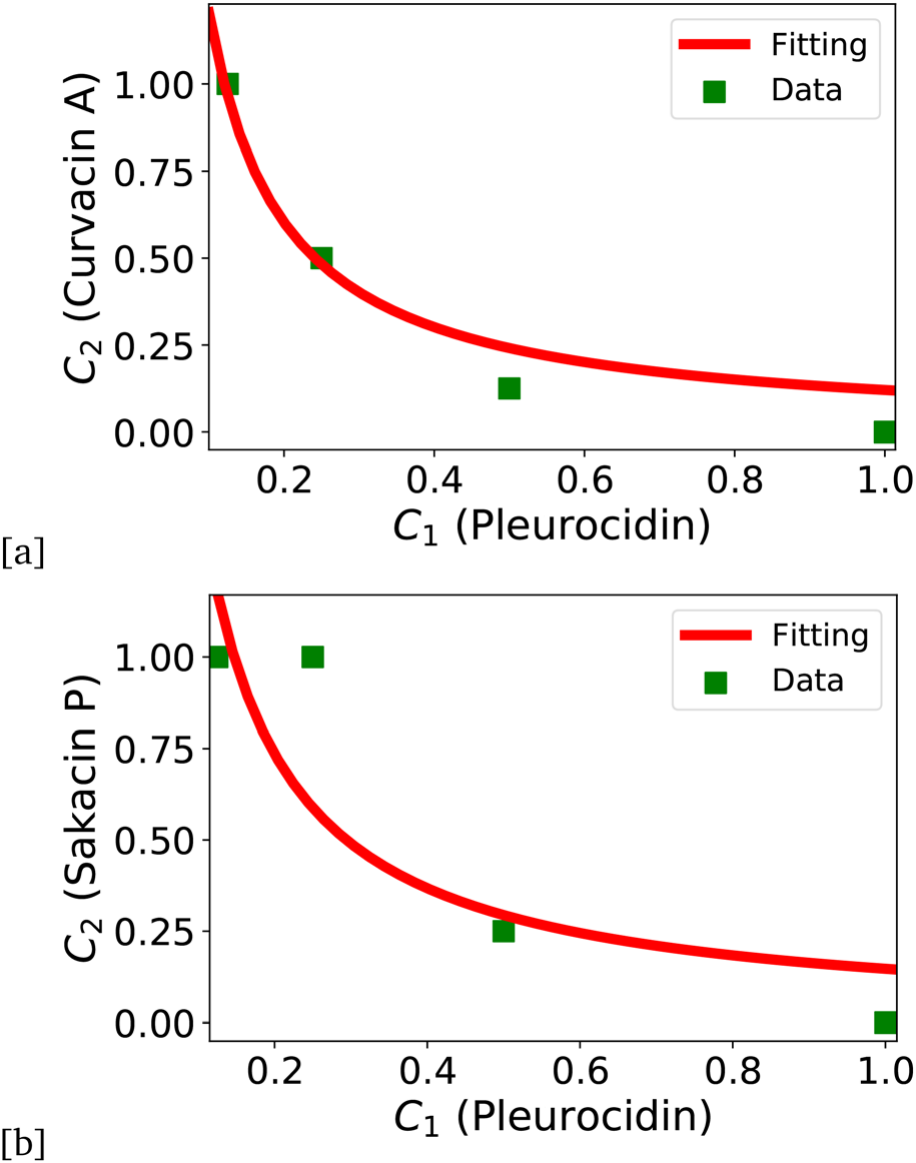
Analysis of experimental data.^77^ (a) MIC curves for the combination of curvacin A and pleurocidin AMPs. (b) MIC curves for the combination of sakacin P and pleurocidin AMPs. The figure is adopted with permission from ref. 69.

This mapping enables estimation of Δ*E* parameter from the experimental data. To illustrate this, consider experiments when the prokaryotic AMPs (sakacin P and curvacin A) were combined with the eukaryotic AMP pleurocidin to exhibit a strong synergistic action against Gram-negative *E. coli* bacterial strains.^77^ Applying the chemical-kinetic analysis to the experimental data (MICs for each AMP in combination *versus* individually), as illustrated in Fig. 4, yields effective interaction energies approximately Δ*E* ⋍ 2.9*k*_B_*T* for curvacin A + pleurocidin (Fig. 4a) and Δ*E* ⋍ 2.1*k*_B_*T* for sakacin P + pleurocidin (Fig. 4b) combinations.^69^ The larger value corresponds to the more synergistic combination, demonstrating that the framework can not only evaluate synergy but also quantify the degree of cooperativity across systems. However, the framework requires multiple values of MIC in combination and individually to calculate interaction energies, while most published studies only provide the lowest values of MIC or only FIC. Accordingly, the framework has only been demonstrated to date on a limited set of experimental observations.

### 4.4. Larger AMP diversity increases synergy

Beyond two-component mixtures, experimental observations indicate that increasing the heterogeneity of AMP combinations, adding more distinct AMP types, can make antibacterial synergy even stronger.^44^ In particular, more heterogeneous mixtures can exhibit lower FIC (stronger synergy) while maintaining similar total peptide concentrations, suggesting that increasing the number of components changes some aspects of cooperative dynamics.

The chemical-kinetic framework provides not only a way to quantify AMPs heterogeneity but also a microscopic explanation for why increasing the number of components strengthens cooperativity.^82^ The central argument is that synergy arises because intermolecular interactions that stimulate faster association, and therefore the extent of synergy should scale with the number of interactions between different types of AMP molecules available in the system. For a mixture with *m* components, the maximal number of intermolecular contacts between different species is expected when the components are present in comparable amounts, *n*_*j*_ ⋍ *N*/*m*. Under this condition, one AMP molecule of a given type interacts with (*N* − *N*/*m*) molecules of other types, and across *m* types this yields an estimate for the maximal number of contacts (MNC),^82^12
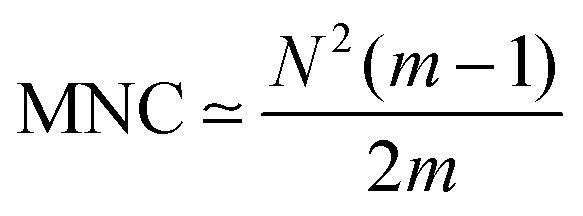


This quantity increases with *m*, rising from *N*^2^/4 for *m* = 2 toward *N*^2^/2 for very large *m*. Thus, increasing heterogeneity increases the number of interactions, accelerating association to bacterial membranes and thereby increasing synergy. In other words, the greater the number of different components in an AMP mixture, the faster the AMPs associate to the membrane, reinforcing the view that synergy and faster association are closely linked.

### 4.5. The role of AMPs' hetero-oligomerization

A closely related phenomenon is the observation that some AMP species can reversibly associate in solution prior to bacterial membrane binding, forming hetero-oligomer species that might also participate in bacterial killing.^88–90^ This raises the question of how oligomerization modifies synergistic activity, particularly because oligomers introduce additional active species and therefore change mixture heterogeneity and association pathways.^91^ The chemical-kinetic framework can be conveniently extended to analyze the effect of reversible oligomerization of AMPs on bacterial eradication cooperativity.^92^

In this approach, the following chemical equations describe the processes in the system,13
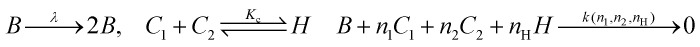
where *H* describes AMP hetero-oligomer molecules, *n*_H_ is the number of such species, and *K*_c_ is the solution equilibrium constant to create hetero-oligomers. Theoretical analysis suggests that reversible oligomerization introduces a new equilibrium-controlled dimension to cooperative behavior.^92^

The application of the chemical-kinetic framework for AMPs systems with hetero-oligomerization indicates that the overall bacterial eradication dynamics are controlled by two competing factors.^92^ First, oligomerization produces new AMP species that also participate in bacterial killing, and from this point of view it corresponds to increasing heterogeneity. It was found that the presence of oligomers makes originally two-component positively cooperating systems effectively three-component and thus more synergistic.^88–90^ Similarly, for originally antagonistic two-component systems oligomerization leads to even stronger negative cooperativity. No effect is observed for originally additive (no interactions) systems.

However, hetero-oligomerization equilibrium adds additional constraints in the system. Although producing more hetero-oligomers initially is beneficial for the system, producing stronger synergy due to increase in the overall AMPs heterogeneity, it becomes a problem when the equilibrium is shifted too much in the direction of hetero-oligomers. In this case, the heterogeneity will start to decrease. These arguments suggest that there are optimal conditions for equilibrium that lead to the largest increase in synergy. The oligomerization effect is strongest at intermediate concentrations where heterogeneity is maximal, *i.e.*, when *C*_1_ ∼ *C*_2_ ∼ *H*. Using the equilibrium relation and normalization, this yields an estimate for the strongest cooperativity when the concentrations of all AMPS are comparable,14
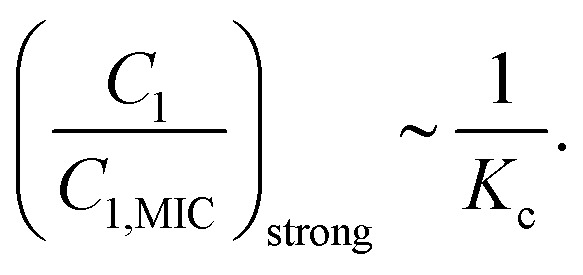


These calculations suggest that hetero-oligomerization can be used as a convenient tool to regulate cooperativity in AMP combinations.

## 5. Predicting synergy: statistical and machine-learning approaches

### 5.1. The combinatorial challenge

To successfully use AMPs in eliminating infections it is not enough to provide microscopic explanations of synergy, but it is also critically important to have specific predictions of AMP combinations against specific targets. One of the central obstacles in exploiting synergistic antimicrobial peptides is the immense size of the combinatorial space. Currently, more than 10^3^ distinct AMPs have been identified and characterized, and this number keeps increasing.^93^ This suggests more than 10^6^ possible pairwise combinations are possible, and the number of combinations with more than two components grows exponentially with the number of different species. Experimentally testing even a small fraction of these combinations is not feasible. This challenge is further complicated by the bacteria-specific nature of synergy. FIC values for AMPs combinations depend strongly on the bacterial species tested, and datasets are typically sparse, heterogeneous, and unevenly distributed across different organisms. As a result, simple pooling of data across bacterial targets obscures meaningful correlations and limits predictive power.^94^ These constraints motivate the development of computational, statistical and machine-learning approaches that can identify likely synergistic combinations and guide experimental prioritization.

### 5.2. Feature-based representation of AMP combinations

To address this challenge, a feature-based statistical framework has recently been introduced.^95^ It represents AMP pairs in terms of their physicochemical properties rather than their sequences alone. In this method, each type of AMP molecule is mapped to a high-dimensional vector of physicochemical descriptors extracted using the *propy* computational tool.^96^ In total, 1547 descriptors were considered, including amino acid composition, net charge, hydrophobicity, polarizability, solvent accessibility, van der Waals interactions, and sequence-dependent autocorrelation functions. An AMP pair (*A*, *B*) is then represented by a single parameter, which is computed as the Euclidean distance, *d*(*A*, *B*), between the two peptides in the multi-dimensional features space. This distance is interpreted as an inverse measure of physicochemical similarity,15
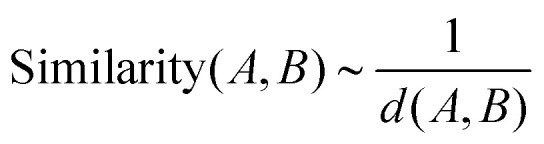


Crucially, the analysis demonstrates that distance computed over all descriptors does not correlate with synergy. Instead, correlations emerge only after feature selection, performed separately for each bacterium by identifying descriptors whose distances exhibit statistically significant Spearman correlations with FIC values (*p* < 0.005). The physical meaning of this is to identify the most important physicochemical properties relevant for interactions between different AMP species. This procedure yields bacterium-specific reduced feature sets, enabling a compact and interpretable representation of AMP complementarity.

### 5.3. Prediction of novel AMP combinations

Statistical and machine-learning analysis using the reduced bacterium-specific feature space, is able to clarify the correlations between the similarity of physicochemical properties and synergy in antibacterial action. For three analyzed bacteria, (*E. coli*, *M. luteus*, *P. aeruginosa*),^95^ synergistic AMP pairs exhibit larger distances in selected physicochemical features than non-synergistic pairs. In other words, AMPs that are more dissimilar (larger *d*(*A*, *B*)) in key properties are more likely to cooperate. In addition, when Euclidean distance is computed using only the selected features, AMP pairs with larger distances systematically correspond to lower FIC values, allowing clear separation between strongly synergistic and non-synergistic combinations.^95^ This separation allows prediction of new synergistic AMP combinations using defined distance thresholds. Distances of 0–0.5 correspond to non-synergistic pairs, whereas distances of 1.5–2.5 indicate synergistic pairs. By calculating the distances between untested peptide combinations and classifying them according to the defined thresholds, pleurocidin was predicted to act synergistically with trout histone H1 (1–26) against *E. coli*. As shown in Table 1, the distance between pleurocidin and H1 falls within the range corresponding to synergistic pairs. Table 1 further shows that this distance is similar to the distances for pleurocidin combined with other peptides, where synergy has been experimentally observed.^95^

**Table 1:** Combinations of pleurocidin with different AMPs, showing predicted or observed synergy against *E. coli*, and the distances in selected physicochemical features between AMPs in each combination. The data were obtained from previously reported analyses in ref. 95

Synergy	AMP 1	AMP 2	Distance
Predicted	Pleurocidin	H1	2.0
Observed	Pleurocidin	PA-1	1.7
Observed	Pleurocidin	Curvacin-A	2.1

### 5.4. The role of amphipathicity in AMP synergy

Only a small subset of descriptors is required to recover these correlations between distance and synergy. While the specific features differ between bacterial species, several classes of universal properties, including hydrophobicity and polarizability, have been identified. Importantly, autocorrelation in hydrophobicity, which reflects the amphipathicity of AMPs, is repeatedly identified as a key discriminating property. Synergistic pairs often consist of one amphipathic peptide and one non-amphipathic peptide, whereas pairs of peptides with similar amphipathic character tend to be non-synergistic.

**Fig. 5:**
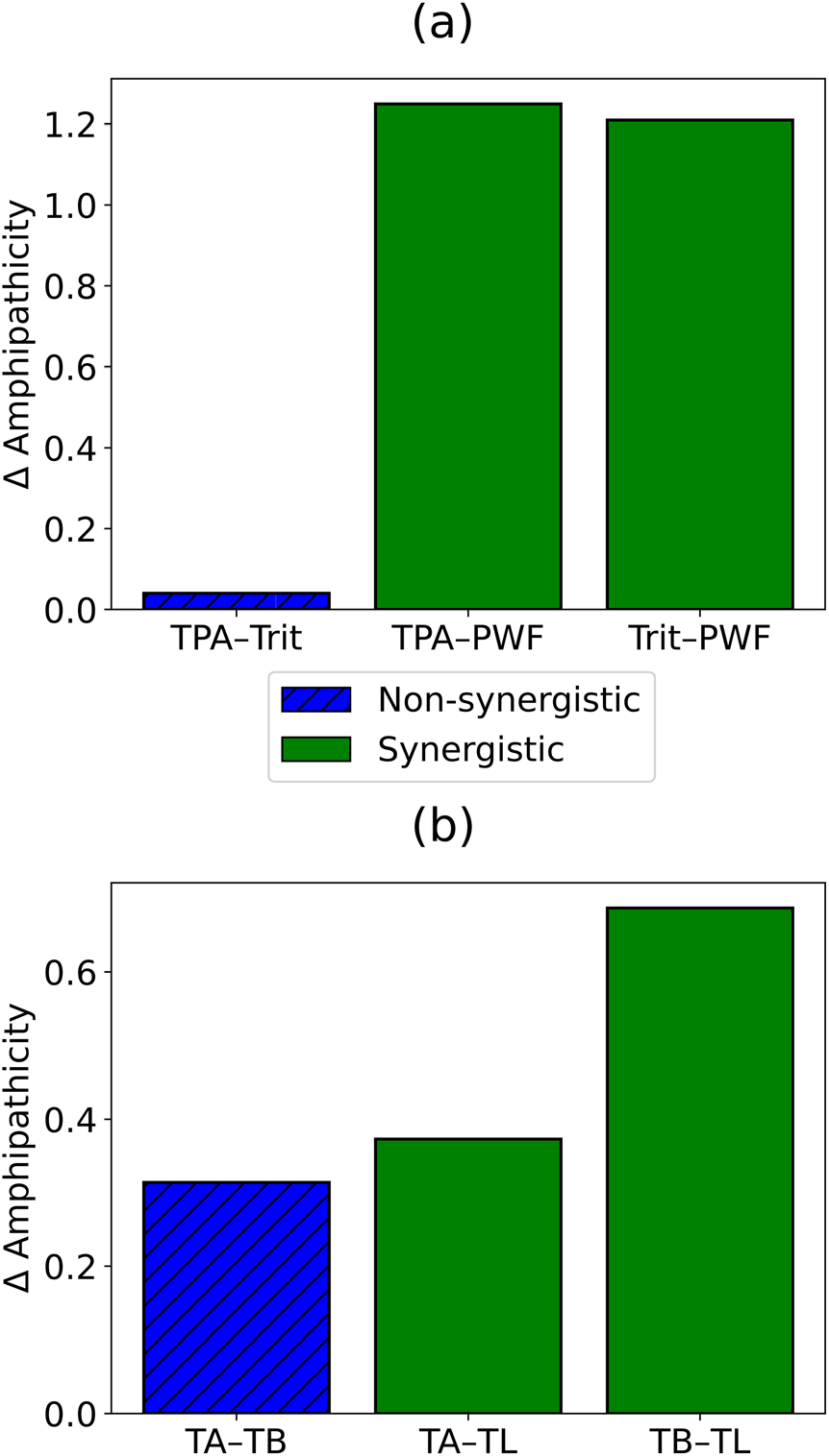
Pairwise differences in estimated amphipathicity between antimicrobial peptide (AMP) pairs in (a) Tritrpticin and (b) Temporin classes. The data were obtained from previously reported analyses in ref. 95. Blue hatched bars represent non-synergistic pairs, and solid green bars represent synergistic pairs.

However, it is not just the absolute amphipathic character that plays a role. Relative differences in amphipathicity also appear to be important. When the same AMP is combined with different partner peptides, the synergistic pair tends to show a larger inter-peptide difference in amphipathicity than the non-synergistic pair. Fig. 5 illustrates these differences by quantifying amphipathicity as the hydrophobicity autocorrelation at long sequence distances (distance 11 in panel a and distance 9 in panel b). The inter-peptide amphipathicity difference for each pair was calculated as the absolute difference in this hydrophobicity feature. In Fig. 5(a), TPA and Tritrpticin (Trit) display small amphipathicity differences and form a non-synergistic pair. In contrast, the amphipathicity difference is nearly a magnitude larger between TPA and PWF, a synergistic pair. The same trend is observed when comparing Trit in combination with PWF *versus* TPA. Similarly, in Fig. 5(b), the synergistic pairs Temporin A (TA)–Temporin L (TL) and Temporin B (TB)–Temporin L (TL) show larger inter-peptide amphipathicity differences than the non-synergistic TA–TB combination. Among these, the TB–TL pair exhibits the largest amphipathicity difference.

These results support a general principle: synergy is associated with physicochemical complementarity rather than similarity, extending the kinetic-cooperativity framework developed in earlier sections of this review by providing a possible basis for positive cooperativity between AMPs. In other words, two different AMP species complement each other in their abilities to kill bacteria, and this leads to strong synergy. However, the complementarity usually means that AMPs have different physicochemical properties, at least those that are crucial for their antibacterial functioning.

## 6. Limitations and open questions

While the chemical-kinetic and statistical frameworks reviewed above provide a comprehensive, coherent and quantitatively consistent picture of synergistic antimicrobial activity, several important limitations and open questions remain. Addressing these issues will be essential for advancing the field by developing better theoretical methods and for more quantitative analysis of experiments.

A central assumption underlying the chemical-kinetic framework is that association of AMPs to the bacterial membrane is the rate-limiting step governing the overall process of bacterial elimination. This assumption is motivated by extensive experimental and theoretical evidences that membrane binding is always the first step in AMP activity and that pore formation or membrane disruption typically follows association. However, this assumption may not hold universally. In some systems, downstream biochemical or biophysical processes, such as pore stabilization, membrane remodeling, intracellular target engagement, or metabolic collapse, might be more important for the overall dynamics of bacterial eradication. If these later steps are rate-limiting, *i.e.*, slower than the AMP membrane association processes, then accelerating membrane binding alone may not translate into enhanced antibacterial efficacy. Consequently, while cooperative membrane association provides a reasonable and testable mechanism for synergy, its relative importance may vary across AMP classes, bacterial species, and environmental conditions.

Another simplifying assumption in the chemical-kinetic approach is that AMP combinations act through the same fundamental mechanisms as individual peptides, differing primarily in the rates at which these mechanisms are engaged. In reality, interactions between different AMP species may open qualitatively new pathways for bacterial elimination. There are some indications that this might be the case in some systems. Experimental studies have suggested that AMP combinations can exhibit behaviors not observed for individual peptides, including altered pore architectures, enhanced membrane destabilization, or coordinated action between membrane-active and intracellular-targeting peptides. Such emergent mechanisms could contribute to synergy independently, or in addition to, accelerated membrane association. At present, the theoretical framework does not explicitly account for the emergence of new biochemical pathways when AMPs act on bacterial species. Incorporating such effects is possible through coupling chemical-kinetic descriptions of association with more detailed mechanistic models of membrane disruption and intracellular activity.

It is also important to emphasize that the chemical-kinetic framework presented above is inherently a mean-field picture of underlying processes, which assumes homogeneous membranes and averaged intermolecular interactions. In biological systems, however, bacterial membranes are highly heterogeneous, with spatially varying lipid composition, curvature, and local charge density. Similarly, AMP binding is inherently stochastic and spatially clustered, leading to local membrane perturbations that may strongly influence subsequent binding events. While some molecular dynamics studies support the idea of cooperative binding near already perturbed membrane regions,^84,85^ these spatial correlations are not explicitly captured in the current framework. Future extensions that incorporate spatial heterogeneity, clustering, and local membrane remodeling could provide a more realistic description of cooperative AMP action and may reveal additional sources of synergy or antagonism.

Another limitation is that in the chemical-kinetic method, effective intermolecular interactions between different AMP species are characterized by a single interaction energy parameter, Δ*E*, often assumed to be similar for all heterotypic pairs within a mixture. This simplification enables analytical progress that clarifies some aspects of the underlying biochemical processes, but is unlikely to hold in real systems, where interaction strengths might depend sensitively on peptide sequences, structures, and environment. Because Δ*E* enters the calculations through exponential factors, there could be quantitative deviations due to noise in experimental measurements. While the qualitative predictions, such as the correspondence between positive Δ*E* and synergy, are expected to remain robust, improved experimental and computational estimates of pair–specific interaction energies will enable more precise predictions.

Despite encouraging agreement with existing experimental data, several validation gaps remain. Most available datasets are sparse, bacterium-specific, limited in dynamic range of FIC values, and often collected under heterogeneous experimental conditions. This limits both kinetic parameter extraction and machine-learning model training. In particular, systematic measurements of membrane association rates, oligomerization equilibria, and synergy metrics for the same AMP combinations under controlled conditions are still uncommon. Bridging this gap is possible through closer integration between theory-driven predictions and targeted experimental design.

## 7. Future directions

**Fig. 6:**
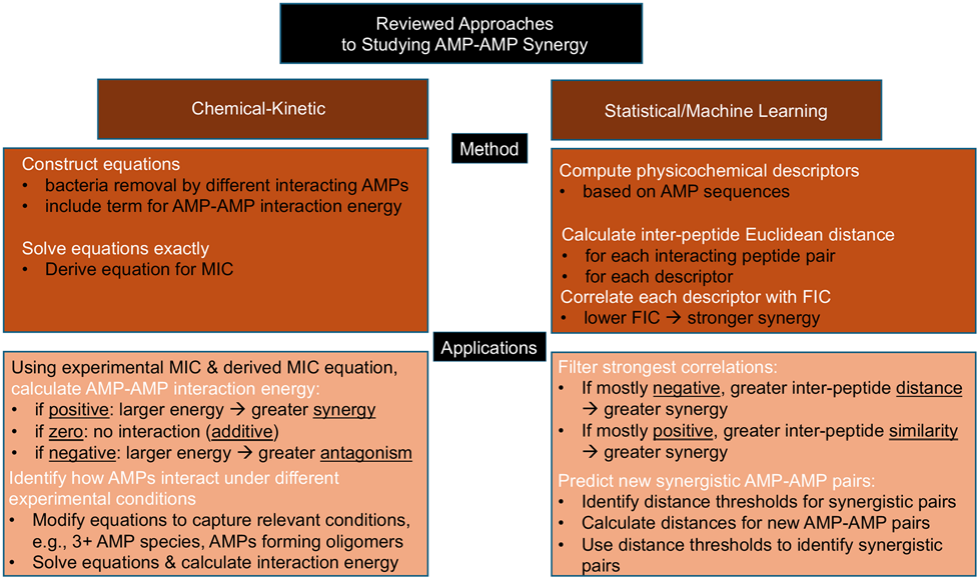
Summary of the theoretical/computational approaches for analyzing antimicrobial peptides discussed in this review.

Significant progress has been made in our understanding of the molecular mechanisms of how AMPs eliminate bacterial species. Despite some limitations, the collective body of work reviewed here outlines a promising and conceptually unified path toward understanding and exploiting synergistic antimicrobial peptide combinations. The corresponding approaches for this path are summarized in Fig. 6.

A central conceptual advance is the identification of Δ*E* as a unifying parameter that links microscopic intermolecular interactions, *via* kinetic acceleration of membrane association, and macroscopic synergy metrics such as MIC and FIC. By mapping experimentally measurable quantities onto an effective free-energy scale, Δ*E* provides a physically interpretable measure of cooperativity that is comparable across systems. The framework demonstrates that relatively weak interactions, on the order of a few *k*_B_*T*, are sufficient to generate substantial synergy due to accelerated association rates of AMP mixtures compared to individual AMP types. At the same time, many questions remain about the heterogeneity and reliability of these parameters in predicting the antibacterial properties of mixtures of AMPs. A better description from both theoretical and experimental points of view should improve our understanding of underlying microscopic processes.

The statistical and machine-learning analyses of physicochemical complementarity provide a useful complementary method to chemical-kinetic framework that allow to make more realistic predictions. Feature-based approaches identify which AMP pairs are likely to exhibit cooperative interactions, while chemical-kinetic models explain why those interactions translate into enhanced antibacterial activity. Together, these approaches suggest a multi-layered strategy that must involve physicochemical descriptors and machine learning to navigate the combinatorial space, chemical-kinetic modeling to interpret synergy mechanistically, and targeted experiments to validate and refine predictions. Such integration can move the field toward predictive, mechanism-informed rational design of future antibiotic therapies.

The reviewed results point to several emerging design principles for AMP combinations. Physicochemical complementarity, rather than similarity, favors synergy. Hetero-oligomerization of AMPs prior to their antibacterial action can also enhance or suppress synergy depending on the underlying cooperativity and must be tuned rather than maximized. Pairwise testing alone may be insufficient, as synergy in multi-component mixtures can change with the number of different components in the mixture. More promising are the combinations of more than two AMPs, but they require additional chemical-kinetic and statistical analysis. These insights suggest that rationally designed AMP combinations could achieve high efficacy at lower total concentrations, reducing toxicity and limiting the emergence of resistance. Finally, AMP combinations offer an efficient strategy for resistance management. By accelerating bacterial killing, engaging multiple cooperative pathways, and exploiting physicochemical complementarity, heterogeneous AMP mixtures may reduce the likelihood of resistance evolution compared to single-agent therapies.

Beyond antimicrobial applications, the conceptual frameworks discussed here, cooperativity through kinetic acceleration, complementarity-driven function, and effective free-energy descriptions, might prove relevant to a wide range of biological systems where collective molecular action determines the function. In this sense, AMP synergy serves not only as a practical therapeutic opportunity, but also as a model system for studying cooperativity at the interface of chemistry, physics, and biology.

## Author contributions

A. M. and A. B. K. jointly conceptualised, wrote, and edited this review.

## Conflicts of interest

There are no conflicts of interest to declare.

## Data Availability

No primary research results, software or code have been included, and no new data were generated or analyzed as part of this review.
